# Tribute to David P. Farrington (1944–2024): Originally published by WestEd Justice & Prevention Research Centre. Used by permission from WestEd. https://jprc.wested.org/from-the-jprc-directors-desk-tribute-to-david-p-farrington-1944-2024/

**DOI:** 10.1002/cl2.70049

**Published:** 2025-06-02

By Anthony Petrosino, Senior Fellow & Affiliated Faculty, Center for Evidence‐Based Crime Policy, George Mason University.

I was saddened to learn of the death of Professor David Farrington of Cambridge University on November 5, 2024. David was a pioneering figure in the field of criminology, whose work and influence spanned decades and left an indelible mark on both academic and practical approaches to crime and justice. He has long been considered one of the most influential criminologists in history. *ScholarGPS* ranks David at the top of its list of criminology scholars for impact, productivity, and quality (see https://scholargps.com/scholars/20828811083920/david-p-farrington). Some of us used to joke that we needed a forklift to move David's printed vita, which was well over 100 pages. There are many wonderful tributes to David and his professional accomplishments, so my comments will focus more on our personal connections (e.g., see J. W. Thulborn's tribute, “David P. Farrington, O. B. E., Distinguished Criminologist and Scholar 1944–2024” at https://www.crim.cam.ac.uk/news/david-p-farrington-obe-distinguished-criminologist-and-scholar-1944-2024. Those who want to better understand David's career pathway, at least through 1997, may appreciate this interview of him by Rolf Leiber for the American Society of Criminology Oral History of Criminology Project at: https://asc41.org/oral-history/david-farrington-interviewed-by-rolf-loeber-november-20-1997/).

One of my first interactions with David came when I was doing my dissertation in the mid‐1990s. I reached out to him, as he was one of the leading proponents of using randomized controlled trials (RCTs) to test criminological interventions (Farrington 1983), and he was excited to hear that my research focused on individual‐level RCTs that tested a strategy to reduce criminal offending (Petrosino 1997). He sent me relevant articles and reports, and periodically would send encouraging notes pushing me to finish the dissertation. Later, after I got the doctorate, I put in a proposal to Oxford University Press for a book based on my dissertation research. I later learned that David was a peer reviewer and the strongest advocate for Oxford to contract with me to write it. Unfortunately, my progress stalled on this book idea when other work commitments got in the way. David pushed me to finish, saying “it was the thing that would last,” and I wish I had listened to him, as I remain “bookless” as an author, all these years later.

We finally crossed paths in person in 1998 when I got involved in preliminary efforts to launch the Campbell Collaboration (Petrosino 2013). Building on the success of its older sibling, the Cochrane Collaboration in health care, the Campbell Collaboration would prepare, update, and disseminate high‐quality reviews of research on the effects of social and educational interventions. Sir Iain Chalmers had been instrumental in launching the Cochrane Collaboration and was working with Professor Robert Boruch of the University of Pennsylvania to lay the groundwork for Campbell. That groundwork included forming coordinating groups and choosing who to lead these groups to oversee systematic reviews in particular policy areas. For such a nascent effort, leaders with great legitimacy in the field and a big network were needed. One of the easiest decisions was that the Campbell Collaboration Crime and Justice Coordinating Group should be led by David Farrington.

Iain and I traveled to Cambridge, where we met David, along with Sharon Cure, who was working on a study of RCTs relevant to aggressive and violent behavior (Cure et al. 2005). David could not have been more generous, not only enthusiastically agreeing to lead the Crime and Justice Group but also inviting me to give a presentation to a plenary session of the 1999 American Society of Criminology (ASC) meeting. This was a big deal. As ASC President at that time, he could form several Presidential plenary sessions of his choosing. These are usually among the best‐attended sessions at the conference, and it was his way of giving the Campbell Collaboration—and me—broad exposure. My knees were knocking to be on a panel chaired by David and including eminent scholars like James Short and David Weisburd, but I got through the presentation. And it had an immediate impact. The wonderful, late Temple University professor, Joan McCord, was among those in the large audience. She heard my presentation and immediately went to meet with Robert Boruch at the University of Pennsylvania to get involved with the Campbell effort, which she vigorously assisted for many years until her death.

David was exactly the leader the Campbell Crime and Justice group needed. Right away, he secured funds to support the group's early activities, including support for me to serve as Coordinator. I worked closely in that role with David until I stepped down in 2004. What an honor for a Jersey boy like me to serve with David on that first Steering Committee which included criminologists from around the globe: Catherine Blaya (France), Ulla Bondeson (Denmark), Vincente Garrido (Spain), Peter Grabosky (Australia), Mark Lipsey (USA), Friedrich Losel (Germany), Joan McCord (USA), Lawrence Sherman (USA), Chuen‐Jim Sheu (China), Richard Tremblay (Canada), Hiroshi Tsutomi (Japan), Brandon Welsh (USA), David Weisburd (Israel), and David Wilson (USA).

As expected, David's reputation helped the group secure funding and commitments from busy researchers to lead the reviews. I was also impressed with how organized he was as a Chair, and how good at facilitating meetings of a lot of smart and talkative folks—no easy task. In fact, at the official launch of the Campbell Collaboration in Philadelphia, the legendary Harvard University statistician, Frederick Mosteller, attended our Crime and Justice Group breakout session. After watching David lead the meeting, Fred, considered a very good chair of meetings himself, remarked, “Now that is the way to run a meeting.”

David was also an engine of scholarly production. We used to kid that he published seven articles before we were done brushing our teeth in the morning. The tributes after his death have credited him with over 800 publications. I once asked him his secret. One, he said, was to have great collaborators as coauthors. Second, he generally wrote for 2 h in the morning before getting involved in the other busyness of the day, especially looking at emails.

As he published, he looked for ways to lift up his junior colleagues and include them as coauthors. Reviewing my own vita, I count 11 academic publications that I was blessed to have coauthored with David (The first was Farrington and Petrosino 2000; the last was Morgan et al. 2021). Anyone who wrote with David would not consider it to be “easy,” however. He was a quick writer and a vociferous editor, and we spent a whole lot of time doing revisions even before submitting to a journal. And he never forgot about me, even after I moved from academic leanings into the research contract world. Every few years, even after I stepped down from the Campbell effort, I would get an invitation from David to contribute to some new collection of articles he was putting together.

Finally, when we launched the new Justice and Prevention Research Center at WestEd in 2014 (that I directed until 2025), we determined to identify key advisors to guide us. The short list included David. Again, he was only too eager to help, and he served in this role until his death. David would periodically email me suggestions on obtaining funding, contribute as an expert advisor on our research grants, and otherwise promote the Center in any way he could.

Graham Nash (of the famous rock band, Crosby, Stills, and Nash) once said, “getting older is not for the faint‐hearted.” He was referring to how you face your own health issues and mortality. But I think one of the most painful things about getting older is losing those wonderful mentors, sages, and guides who have enriched your life, those who helped prepare you, lifted you up, and made your life a little bit better. I am sad that we lost David. But I am grateful that there are generations of researchers—including me–celebrating him for all he has done for the field and our careers.
